# pHisPred: a tool for the identification of histidine phosphorylation sites by integrating amino acid patterns and properties

**DOI:** 10.1186/s12859-022-04938-x

**Published:** 2022-09-28

**Authors:** Jian Zhao, Minhui Zhuang, Jingjing Liu, Meng Zhang, Cong Zeng, Bin Jiang, Jing Wu, Xiaofeng Song

**Affiliations:** 1 Department of Biomedical Engineering, Nanjing University of Aeronautics and Astronautics, Nanjing, 210016 China; 2 College of Automation Engineering, Nanjing University of Aeronautics and Astronautics, Nanjing, 211106 China; 3 School of Biomedical Engineering and Informatics, Nanjing Medical University, Nanjing, 211166 China

**Keywords:** Histidine phosphorylation, Phosphohistidine site, Machine learning, pHis prediction, pHisPred

## Abstract

**Background:**

Protein histidine phosphorylation (pHis) plays critical roles in prokaryotic signal transduction pathways and various eukaryotic cellular processes. It is estimated to account for 6–10% of the phosphoproteome, however only hundreds of pHis sites have been discovered to date. Due to the inherent disadvantages of experimental methods, it is an urgent task for developing efficient computational approaches to identify pHis sites.

**Results:**

Here, we present a novel tool, pHisPred, for accurately identifying pHis sites from protein sequences. We manually collected the largest number of experimental validated pHis sites to build benchmark datasets. Using randomized tenfold CV, the weighted SVM-RBF model shows the best performance than other four commonly used classification models (LR, KNN, RF, and MLP). From ten thousands of features, 140 and 150 most informative features were individually selected out for eukaryotic and prokaryotic models. The average AUC and F1-score values of pHisPred were (0.81, 0.40) and (0.78, 0.46) for tenfold CV on the eukaryotic and prokaryotic training datasets, respectively. In addition, pHisPred significantly outperforms other tools on testing datasets, in particular on the eukaryotic one.

**Conclusion:**

We implemented a python program of pHisPred, which is freely available for non-commercial use at https://github.com/xiaofengsong/pHisPred. Moreover, users can use it to train new models with their own data.

**Supplementary Information:**

The online version contains supplementary material available at 10.1186/s12859-022-04938-x.

## Background

Protein phosphorylation, a reversible mechanism of posttranslational regulation, is critically important in most cellular processes including cell cycle, growth, apoptosis, and signal transduction pathways [1]. Of the 20 basic amino acids, nine can be phosphorylated. The most known phosphoamino acids are serine (Ser), threonine (Thr), and tyrosine (Tyr), which accounts for the majority of phosphorylation events. The histidine (His) residues in proteins also can be phosphorylated, which was once assumed to be rare in cells [2]. Recent studies, however, show that histidine phosphorylation is more common than previously thought, and it may account for as much as 6% of total eukaryotic phosphoamino acids [3–5].

Histidine phosphorylation has been extensively reported in prokaryotic signal transduction pathways, particularly in the bacterial two-component regulatory systems (TCS) and phosphotransferase system (PTS) [6, 7]. A recent mass spectrometry-based proteomics study proved that prokaryotic histidine phosphorylation is widespread and abundant, and phosphohistidine (pHis) accounts for a remarkably high percentage (~ 10%) of the phosphoproteome [3]. In contrast, eukaryotic histidine phosphorylation remains largely unexplored and only two histidine kinases (NME1 and NME2) are well-investigated [8–10]. While not as well-characterized in higher eukaryotes, histidine phosphorylation has been detected in a variety of cellular processes including signal transduction, proliferation, differentiation, development, apoptosis, cytokinesis, and dynamin-mediated endocytosis [11–13]. Recent evidence implicates that pHis signaling is involved in cancer and tumor metastasis [14–16].

Histidine phosphorylation sites are much less well recognized than Ser, Thr, and Tyr phosphorylation sites, despite histidine phosphorylation having been first discovered about 60 years ago [17]. A major reason is that histidine phosphate linkage is labile in acidic pH conditions, which reduced pHis half-life and make is hardly to be detected by the commonly used protocols in LC–MS/MS analysis [18–20]. Over the last few years, due to the development of histidine phosphate analogs and pHis monoclonal antibodies, diverse experimental methods have emerged for the detection of pHis sites and have led to a resurgence in the study of histidine phosphorylation [21]. To date, hundreds of novel pHis sites have been discovered in both eukaryotes (e.g., *Homo sapiens*, *Danio rerio*, and Bos taurus) and prokaryotes (e.g., *E. coli*) [3, 22–25].

The inherent disadvantages of experimental methods, however, make it an urgent task for developing efficient computational approaches to identify pHis sites, which can reduce the cost and time of experimental methods and provide a useful validation for biological experiment. There have been many computational predictors (e.g., GPS, MusiteDeep, DeepPhos, and PPSP) for identifying phosphorylation sites [26–30], while only few ones were developed specifically for pHis sites. Awais et al. proposed a computational model, iPhosH-PseAAC, using hundreds of pHis sites collected from the UniProt and dbPTM databases as benchmark data [31]. But, to our knowledge, only no more than 70 pHis sites are annotated with publications in UniProt and dbPTM, while most of the pHis sites are predicted according to several specific rules [32, 33]. As such, the predicting accuracy of iPhosH-PseAAC is questionable. Chen et al. used 244 pHis sites of *E. coli* validated by proteomic assay and developed an ensemble model, PROSPECT, for predicting pHis sites in bacteria [34]. This method showed a relatively good classification performance, but challenges remain for accurately identifying prokaryotic pHis sites. Moreover, it is unclear that whether PROSPECT can be applied for predicting eukaryotic pHis sites.

In this study, we developed a new powerful tool named pHisPred based on weighted support vector machine (WSVM) with radial basis function (RBF) kernel. In our benchmark dataset, the pHis sites were manually collected from literature and databases, and they were all validated by experimental methods. Numerous informative features were calculated for the local sequences around pHis and non-pHis sites, and further several different local sequence lengths were tested. Besides SVM, other four types of machine learning (ML) methods were also used for training classification models. Based on the performance of tenfold cross-validation, the optimal combination of local sequence length, feature set, and ML method were obtained to build classification models respectively for eukaryotes and prokaryotes. Compared with PROSPECT, pHisPred performed much better both on the eukaryotic and prokaryotic testing datasets. Finally, we implemented a python program of pHisPred, which is user-friendly and easy to use.

## Results and discussion

### Sequence analysis

**Fig. 1:**
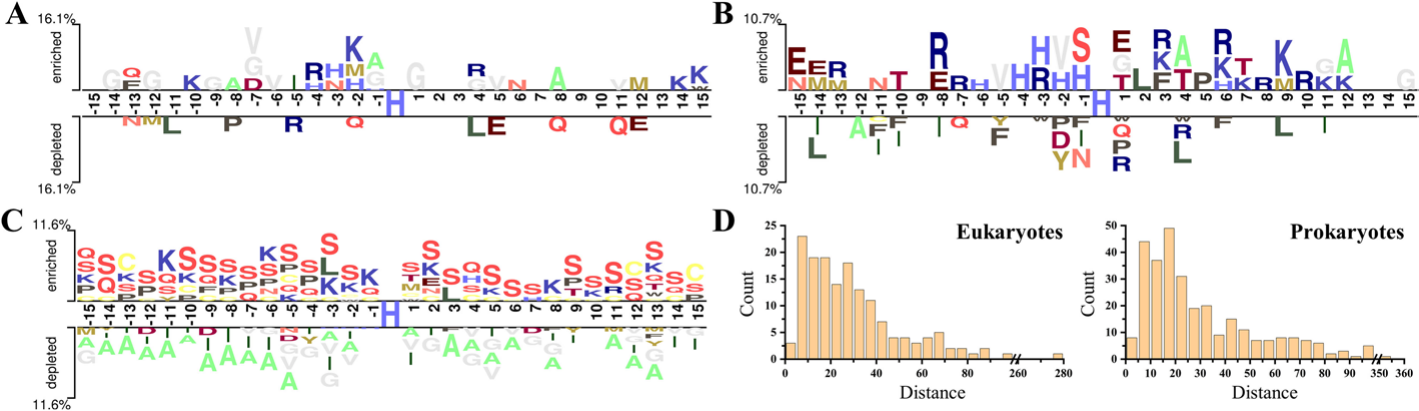
Sequence characteristics of pHis sites in eukaryotes and prokaryotes. **A** and **B** Illustrate the significantly enriched and depleted amino acid residues around the eukaryotic and prokaryotic pHis sites, respectively. **C** The significantly enriched and depleted amino acid residues around eukaryotic pHis sites comparing with prokaryotic pHis sites. **D** The distributions of the sequential distances between pHis and non-pHis sites within the same protein sequences

For the pHis and non-pHis sites, we analyzed the occurrence frequencies of amino acids at each position of their flanking segments. Two Sample Logo with *t-test* (*p* *value* < 0.05) was used to determine the enriched and depleted amino acids flanking the eukaryotic and prokaryotic pHis sites [35]. As shown in Fig. 1A, Glycine (G) shows a higher occurrence frequency on the left and right sides (− 1 and + 1) of the central eukaryotic pHis site. His (H) is slightly enriched at the upstream positions (− 4 to − 2), while the other enriched amino acids are relatively scattered. As shown in Fig. 1B, H is significantly enriched at the left-nearest positions (− 4 to − 1) of the central prokaryotic pHis site, and Lysine (K) shows a slight enrichment at the downstream positions (+ 3 to + 12). Although the eukaryotic segments show less differentially used amino acids than the prokaryotic segments, the amino acid usage bias in some positions of the eukaryotic segments is larger than that in prokaryotic ones.

To investigate whether the eukaryotic and prokaryotic protein sequences could indeed possess differential amino acid preference, we compared the segments flanking the eukaryotic and prokaryotic pHis sites. As can be observed from Fig. 1C, there is an obvious amino acids usage bias between the segments around eukaryotic and prokaryotic pHis sites. The eukaryotic segments prefer to contain more serine (S), lysine (K), proline (P), glutamine (Q), and cysteine (C), while the prokaryotic segments prefer to contain more alanine (A). Interestingly, most of the preferred amino acids (S, K, Q, and C) flanking the eukaryotic pHis sites can be phosphorylated, and S is the most commonly phosphorylated amino acid.

As shown in Fig. 1, the preference of His at the upstream positions flanking the central pHis sites leads to an overlap between positive and negative samples, which would increase the difficulty of distinguishing pHis sites from non-pHis sites. To further examine this overlap, we thus analyzed the statistical distribution of the sequential distances between pHis sites and their nearest non-pHis sites within the same protein sequences (Fig. 1D). The distributions across the eukaryotic and prokaryotic datasets have a similar tendency, with about 26% of the sequential distances being less than 15 amino acids long. This observation indicates that there exists a considerable overlap between the positive and negative samples and further highlights the influence of the segment size on the classification performance when training models.

### Performance evaluation of different feature subsets

**Fig. 2:**
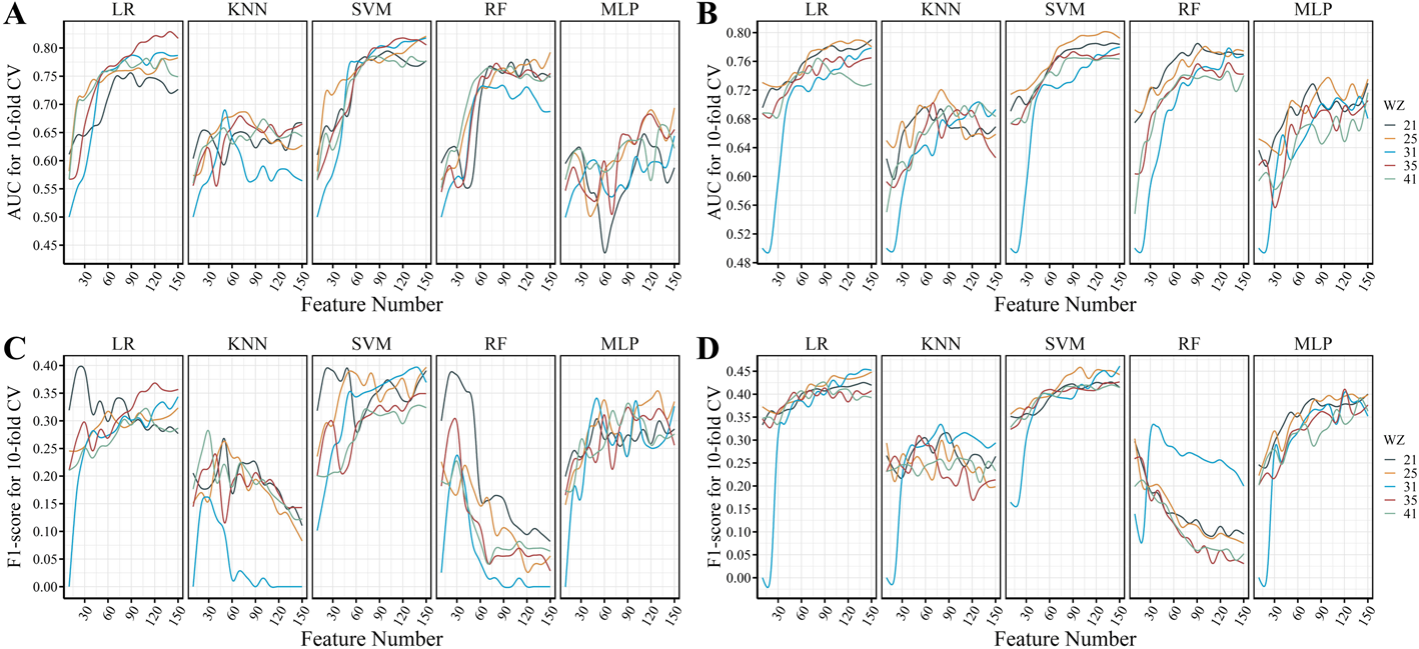
Performance of classifiers with different combinations of model, window size, and feature set. **A** and **C** The average AUC and F1-score of the tenfold cross validation on the eukaryotic training datasets, respectively. **B** and **D** The average AUC and F1-score of the tenfold cross validation on the prokaryotic training datasets, respectively

To select an optimal feature sets for accurate prediction of pHis sites, we tested 150 kinds of feature subsets with five different window sizes using tenfold cross-validation on the eukaryotic and prokaryotic training datasets. With the increase of the window size (21, 25, 31, 35, and 41) used for extracting flanking segments, the dimension of feature vector ranges from 26,368 to 37,326 (Additional file 1: Table S1). For each window size, features with zero variance were removed firstly, and then a specific number of features with the best F-score was selected for training five different models. To find out the optimal number, the values range from 5 to 150 with interval 5 were all tested (Fig. 2).

In most cases, the classification performance (AUC and F1-score) initially increases with the number of features, and eventually become relatively stable without being affected by newly added features (Fig. 2). For different learning models, the best AUC and F1-score were achieved with different window size and feature sets. The window size seems to have a slight influence on the classification performance, especially for the prokaryotic dataset, which contains more sample data. And the optimal window sizes (31 and 35) for the eukaryotic models are larger than that (21 and 25) for the prokaryotic dataset. Unlike the other models, the RF model’s F1-score shows an opposite trend as the number of features increases. Among the five types of models, LR and SVM show the best performance.

As shown in the Fig. 2A and C, on the eukaryotic training dataset, the optimal combinations of feature set and window size for the LR, KNN, SVM, RF, and MLP models are 120 (WZ: 35), 50 (WZ: 25), 150 (WZ: 25), 20 (WZ: 21), and 120 (WZ: 25), respectively (Additional file 1: Table S2). The LR and SVM models achieve the best classification performance. The AUC and F1-score of the LR model are 0.82 and 0.37, respectively; while the AUC and F1-score of the SVM model are 0.82 and 0.40, respectively. As shown in Fig. 2B and D, on the prokaryotic training dataset, the optimal combinations for the LR, KNN, SVM, RF, and MLP models are 150 (WZ: 31), 80 (WZ: 31), 100 (WZ: 25), 130 (WZ: 31), and 100 (WZ: 25), respectively (Additional file 1: Table S3). Similarly, the LR and SVM models achieve the best classification performance. The AUC and F1-score of the LR model are 0.78 and 0.45, respectively; while the AUC and F1-score of the SVM model are 0.80 and 0.46, respectively.

### Performance evaluation of different models

**Fig. 3:**
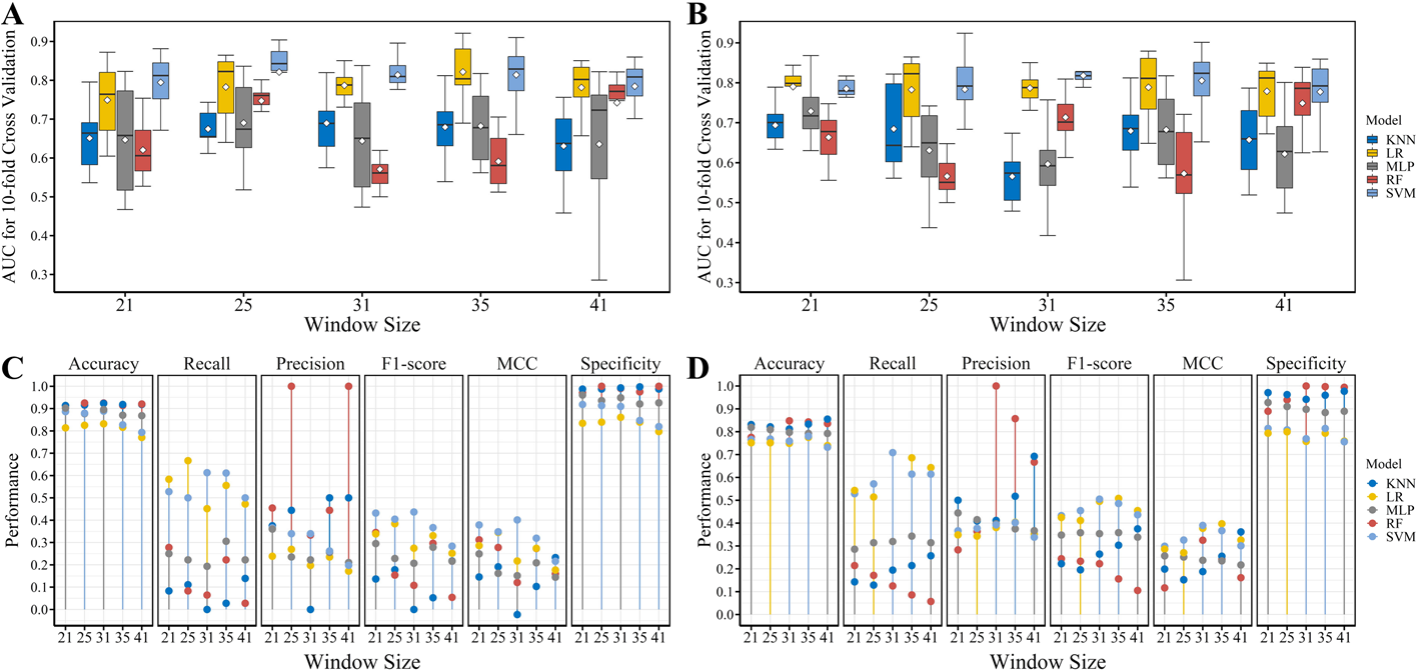
Performance of models with optimal feature set in each window size. **A** and **B**: the AUC values of tenfold cross validation on the eukaryotic and prokaryotic training datasets, respectively. The white dot in each bar represents the average AUC value. **C** and **D** The major performance metrics calculated on the eukaryotic and prokaryotic testing datasets

By using the optimal feature set of the five window sizes, we first built models based on different machine learning methods and compared their performance by performing tenfold cross-validation on the training datasets. As shown in Fig. 3A and B, using either window size, the LR and SVM models all achieve relatively better performance than the other models. And comparing with LR, SMV performs the best in most of the cases. On the eukaryotic dataset, it can be observed that the average AUC scores of the SVM models (WZ: 25, 31, and 35) and the LR models (WZ: 31 and 35) are obviously better than other models, but there is no significant difference between them. The remarkable difference between them is in the distribution of AUC score calculated with the tenfold cross-validation. The SMV model with window size of 31 outperforms other models as it has the minimum variance of AUC score. On the prokaryotic dataset, all the LR and SVM models have similar performance as measured by the average AUC score. However, if measured by the variance of AUC score derived from cross-validation, the SVM model with window size of 21 shows the best performance.

Next, to further compare the above models, we investigated their performance on the testing datasets. As shown in Fig. 3C and D, all the models achieve a good performance in the classification accuracy, even those models with lower AUC score on the training datasets (Fig. 3A, B). This mainly attributes to the imbalance of the testing datasets, which are dominated by negative samples. It can be observed that the models prefer to have good performance in the specificity and poor performance in the recall (also called sensitivity). In addition, the class imbalance also leads to an illusion of the high precision of some models, as only few samples were classified into the positive class. Thus, F1-score and MCC were mainly used to measure the models’ performance.

The SVM and LR models show the best performance in the recall, F1-score, and MCC (Fig. 3C, D). Under most of the window sizes, the SVM models outperform the corresponding RF models. On the eukaryotic testing dataset, the SVM models with window sizes of 21 and 31 show the best performance in the F1-score and MCC, and their classification performances are nearly equal in all the metrics. However, the previous results show that under different feature sets, the SVM model with window size of 31 commonly outperforms that with window size of 21 (Fig. 2A, C). Thus, the SVM algorithm is chosen for building the eukaryotic classification model with 140 selected features, using the window size of 31.

On the prokaryotic testing dataset, the SVM models with window sizes of 31 and 35 show the best performance in the F1-score and MCC. In addition, there are no significant differences between their performance in any of the metrics, except for the recall value. The window size of 31 performs slightly better in the recall, and the window size of 35 performs slightly better in the specificity and accuracy. Although the previous results show that under different feature sets, the SVM model with window size of 25 commonly outperforms that with window size of 31 (Fig. 2B, D), the SMV model with window size of 31 achieves the best performance on the training and testing datasets. Therefore, the SVM algorithm is also chosen for building the prokaryotic classification model with 150 selected features, using the window size of 31.

The same window size and machine learning method are chosen for building both the eukaryotic and prokaryotic classification models; however, the selected feature subsets are almost completely different from each other. Between the eukaryotic and prokaryotic classification models, there are only 14 commonly used features in them (Additional file 1: Table S4). As shown in Table 1, for most of the feature groups, the eukaryotic and prokaryotic classification models show different feature usage frequency. For example, 41 tripeptide composition (TPC) features were chosen for eukaryotic model, while only 6 TPC features were used in prokaryotic model. Although comparable number of AA-index features were used in the eukaryotic and prokaryotic models, the commonly used features account for less than 12%. These results indicate that the eukaryotic histidine phosphorylation is different from the prokaryotic histidine phosphorylation, which is consistent with the observation in the Fig. 1.

**Table 1:** A full list of features used in building classification models

Feature groups	Descriptor	Models	
		Eukaryotic	Prokaryotic
Amino acid composition	Amino acid composition (AAC)	–	1
	Enhanced amino acid composition (EAAC)	1	10
	Composition of *k*-spaced amino acid pairs (CKSAAP)	8	4
	Dipeptide composition (DPC)	3	1
	Tripeptide composition (TPC)	41	6
Grouped amino acid composition	Grouped amino acid composition (GAAC)	1	2
	Enhanced grouped amino acid composition (EGAAC)	–	10
	Composition of k-spaced amino acid group pairs (CKSAAGP)	6	18
	Grouped dipeptide composition (GDPC)	2	2
	Grouped tripeptide composition (GTPC)	–	1
C/T/D	Composition (CTD-C)	1	11
	Distribution (CTD-D)	–	1
	Transition (CTD-T)	–	11
Conjoint Triad	Conjoint Triad (C-Triad)	2	2
	Conjoint k-spaced Triad (CKS-Triad)	2	2
Quasi-sequence-order	Sequence-order-coupling number (SOC-Number)	–	2
	Quasi-sequence-order descriptors (QS-Order)	1	4
Pseudo-amino acid composition	Pseudo-amino acid composition (PAAC)	1	1
	Amphiphilic PAAC (APAAC)	1	-
Autocorrelation	Normalized Moreau-Broto (NM-Broto)	–	–
	Moran	1	–
	Geary	1	–
Binary	Binary	3	5
AA-index	AA-index	64	52
BLOSUM62	BLOSUM62 matrix	–	4
Z-scale	Z-scale	1	–

The details of features used in pHisPred can be seen in Additional file 1: Table S4

### Performance comparison with other state-of-art tools

Since the majority of known pHis sites were experimentally validated in recent years, currently only two tools exist for predicting pHis sites. The earliest tool, iPhosH-PseAAC, was trained using pHis sites collected from the Uniprot and dbPTM databases [31], while the most recent tool, PROSPECT, was trained using pHis sites of *E. coli* derived from a recently published proteomic assay [34]. The source codes of these two methods are not available, and both are provided as a webserver for users. As the website of iPhosH-PseAAC was not available, we only compared our method (pHisPred) with the recent tool PROSPECT. Considering the potential overlap between our prokaryotic testing dataset and the PROSPECT’s training dataset, we removed those samples derived from the same published assay to make a fair comparison.

**Fig. 4:**
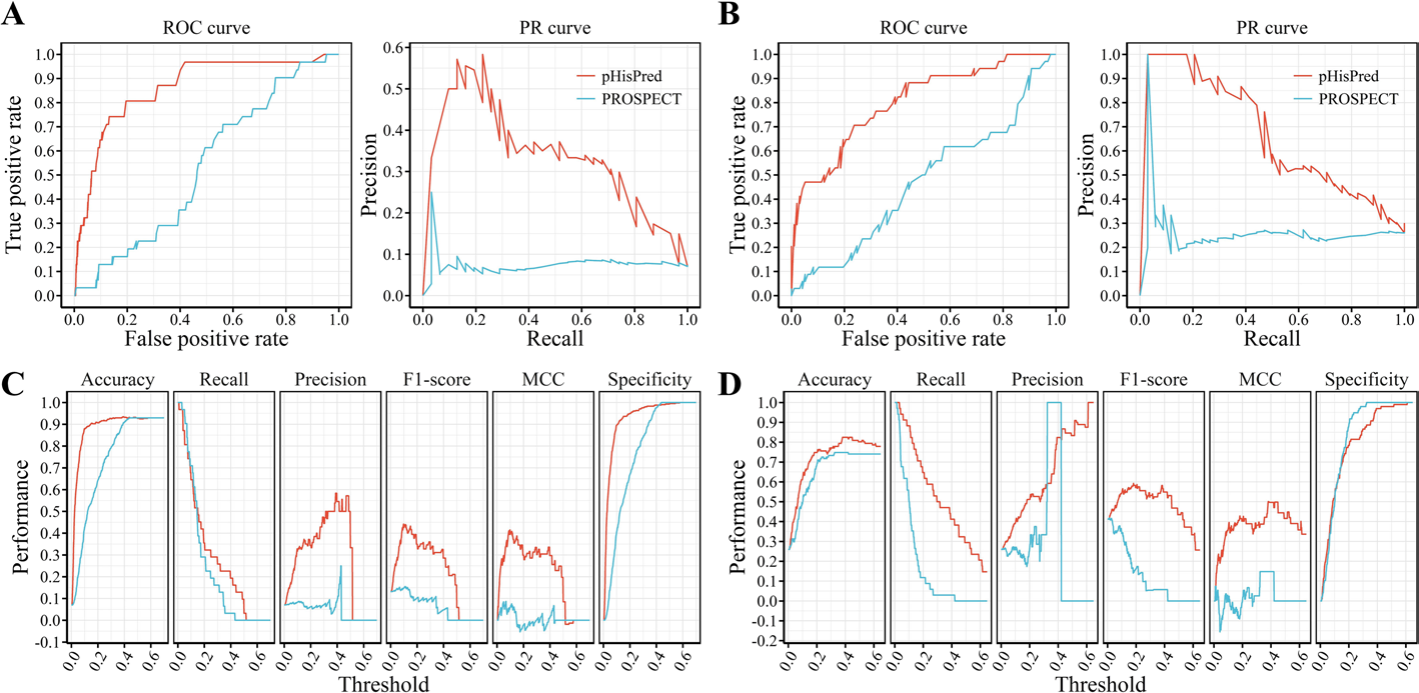
Performance comparison between our proposed method pHisPred and the state-of-the-art method PROSPECT for predicting pHis sites. **A** and **B** ROC and RP curves of both methods on the eukaryotic and prokaryotic testing datasets, respectively. **C** and **D** The major performance metrics vs. classification threshold on the eukaryotic and prokaryotic testing datasets, respectively

To compare the comprehensive performance of pHisPred and PROSPECT, we plotted their ROC curves. On the eukaryotic testing dataset, the area under the ROC curve of pHisPred is much larger than that of PROSPECT (Fig. 4A). The AUC of pHisPred is 0.86, while that of PROSPECT is only 0.53. In addition, we also plotted their precision-recall (PR) curves, because the ROC curve with the imbalanced dataset may not work as well as with the balanced one. As shown in Fig. 4A, pHisPred shows a much better tradeoff between precision and recall for different thresholds than PROSPECT. The F1-score of pHisPred is 0.44, while that of PROSPECT is only 0.13. Besides the ROC curve and the PR curve, we also compared these two methods with respects of several major metrics, including accuracy, recall, precision, F1-score, MCC, and specificity (Additional file 1: Table S5 and S6). As these metrics are directly related to the threshold (or cutoff) value, we plotted threshold-metric curves to objectively perform the comparison (Fig. 4C). It can be observed that pHisPred clearly outperforms PROSPECT on all metrics. The worse performance of PROSPECT indicates that the model trained with prokaryotic data is not suitable for predicting eukaryotic pHis sites, as the usage bias of amino acids between eukaryotic and prokaryotic protein segments (Fig. 1C).

Besides the eukaryotic testing dataset, pHisPred also outperforms PROSPECT on the prokaryotic testing dataset. As shown in Fig. 4B, both ROC and PR curves of pHisPred clearly lie above that of PROSPECT. The AUC of pHisPred is 0.80, while that of PROSPECT is only 0.47; the F1-score of pHisPred is 0.58, while that of PROSPECT is 0.27. Unlike the results on the eukaryotic testing dataset, PROSPECT shows similar performance on the classification accuracy and specificity with pHisPred on the prokaryotic testing dataset. However, on the other metrics (recall, precision, F1-score, and MCC), PROSPECT performs worse than pHisPred (Fig. 4D). This result indicates that pHisPred is better at correctly classifying the positive samples (pHis sites). And the poor performance of PROSPECT may be due to the limited training dataset, which only contains pHis sites from a single species—*E. coli*.

### Availability of pHisPred

To facilitate the community-wide prediction of pHis sites, we have implemented a python program of pHisPred based on the weighted SVM-RBF model with the optimal combination of window size and feature set. In pHisPred, there are two optional classification models respectively for predicting eukaryotic and prokaryotic pHis sites. The source code of pHisPred can be accessed at https://github.com/xiaofengsong/pHisPred, and it is easy to operate and can be performed on any platform. In addition, pHisPred also allows users to train their own models with new data. In the future, with the increase of newly validated eukaryotic or prokaryotic pHis sites, we will update the corresponding classification model in the pHisPred.

## Conclusions

Although with a lot of research interest in pHis functions, there is lack of bioinformatic tools for identifying pHis sites, in particular the eukaryotic ones. Therefore, we curated a data set of histidine phosphorylation sites and developed a novel computational tool—pHisPred, using carefully selected informative features and weighted SVM model with RBF kernel. pHisPred contains two classification models respectively for eukaryotic and prokaryotic proteins, and it is the first tool designed for eukaryotic pHis identification. Moreover, pHisPred outperforms other existing tools on the classification of prokaryotic His sites. pHisPred should aid future pHis studies, especially efforts to elucidate the functions of histidine phosphorylation.

## Material and methods

### Overview

**Fig. 5:**
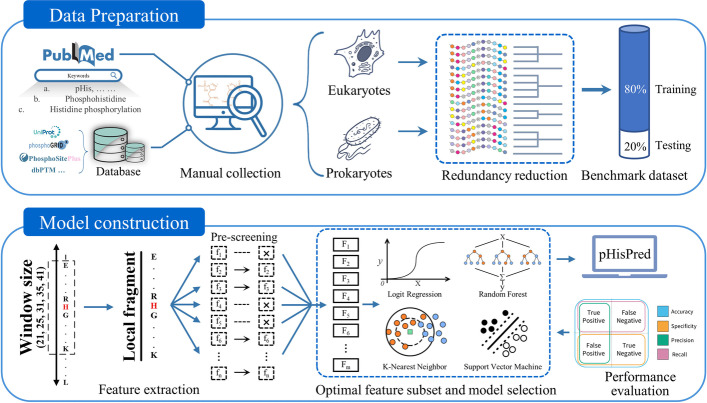
The overall framework of this study. The top panel outlines the process of constructing eukaryotic and prokaryotic datasets. In total, 560 verified pHis sites with experimental evidence were manually collected as positive samples, and 7233 non-pHis sites from the same protein were extracted as negative samples. Based on the local sequences (31 aa) flanking His sites, BLASTCLUST and CD-HIT were used to reduce the data redundancy. The bottom panel illustrates the detailed procedures for constructing pHisPred. Five window sizes were used to extract local sequences flanking His sites. For each window size, ten thousands of features were calculated. Features with constant values were removed. Based on the performance evaluation, the optimal combinations of window size, feature number, and model were individually selected to build eukaryotic and prokaryotic classification models in pHisPred

pHisPred is a novel computational tool for histidine phosphorylation site prediction, and the working flow for developing pHisPred is described in Fig. 5. First, all verified pHis sites supported by experimental evidence were used to construct the benchmark dataset. The dataset construction includes data collection and pre-processing (top panel). The following procedures consist of local sequence extraction, feature calculation, feature pre-screening, selection of the optimal combination of feature subsets and models, performance evaluation, and application of pHisPred. Finally, from hundreds of combinations, the weighted SVM-RBF model with 140 features and 31 window size was selected in pHisPred for identifying eukaryotic pHis sites, while the same model with 150 features and 31 window size was selected for identifying prokaryotic pHis sites. The details were presented in the following sections.

### Benchmark datasets

The high-quality pHis data were mainly collected from published literature (Fig. 5). PubMed search was used with keywords: pHis, phosphohistidine, histidine phosphorylation, phosphorylated histidine, and histidine phosphoproteome [36]. From the retrieved literature, we manually screened out those with pHis verification experiments, and totally found 494 pHis sites after removing duplicates. Besides literature, we also collected 66 pHis sites with experimental evidence from UniProt, dbPTM, PhosPhoSitePlus [37], and other known databases of phosphorylation [38–40]. In final, 560 experimentally validated pHis sites were gathered to construct our dataset, in which 172 pHis sites come from eukaryotic proteins and 388 pHis sites come from prokaryotic proteins.

To build benchmark datasets, the pHis sites were used as positive samples, while the other His sites (non-pHis sites) in the same histidine-phosphorylated proteins were used as negative samples. A sliding window approach was used to extract local sequences centered around the pHis and non-pHis sites from corresponding protein sequences, which were downloaded from the UniProt database. The window size was set to 31, and if the left or right flanking sequences of His sites were short than 15 residues, the missing positions were filled with dummy amino acid—‘X’. To construct non-redundant datasets, the BLASTCLUST program (− L 0.9 and − S 0.9) [41] and CD-HIT (− c 0.9) [42] were performed to reduce the number of samples with similar local sequences. Considering the possible amino acid usage bias in eukaryotes and prokaryotes, we classified samples into two groups and built two distinct benchmark datasets: the eukaryotic dataset (151 pHis sites and 2061 non-pHis sites) and the prokaryotic dataset (336 pHis sites and 1726 non-pHis sites). For each dataset, 80% samples were randomly selected as training dataset, and 20% were remained as testing dataset.

### Feature extraction

The local sequence context surrounding His sites is considered to harbor the most relevant information for the pHis site prediction (Fig. 5). To investigate the optimal relevant information, we extracted a series of His centered local sequences with different length (21, 25, 31, 35, and 41). A dummy residue—‘X’ was used to fill the flanking blank when the actual local sequence is shorter than the specific size. With these local segments centered around His, we extracted numerous informative features using the *iFeature* tool [43]. These features are mainly divided into 11 groups, including amino acid composition, grouped amino acid composition, C/T/D, conjoint triad, quasi sequence order, pseudo amino acid composition, autocorrelation, binary, AA-index, BLOSUM62, and Z-scale.

### Feature normalization and selection

As shown in Table 1, 26 feature encoding methods were applied to generate feature vectors. Due to the difference in encoding strategies, features are shown to have different scales. We note the difference in feature scales will affect the convergence of the gradient descent-based algorithms (e.g., logistic regression and neural network) and impair the classification performance of distance-based algorithms (e.g., KNN, K-mean, and SVM). Therefore, to address this, the *normalize* class in the Python module ‘*sklearn.preprocessing*’ was used to normalize the original values of each feature to the [0, 1] range.

Heterogeneous features extracted from different perspectives might be useful for characterizing pHis sites; however, too many features will also lead to ‘over-fitting’ that can undermine model performance. To reduce the dimension of the feature vector, we employed the class *SelectKBest* in the Python module ‘*sklearn.feature_selection*’. *f_classif* was used as the score function to calculate the ANOVA *F-score* for each feature. The *F-score* is defined as

1 $$SSA = \sum\limits_{i = 1}^{2} {n_{i} (\overline{X}_{i} - \overline{X})^{2} }$$

2 $$SSE = \sum\limits_{i = 1}^{2} {\sum\limits_{j = 1}^{{n_{i} }} {(X_{ij} - X_{i} )^{2} } }$$

3 $$F{ - }score = \frac{SSA}{{SSE/(n - 2)}},$$

where *SSA* denotes the sum of square between pHis and non-pHis sites and SSE denotes the sum of square error for all the His sites. To identify useful feature subsets from the initial feature set, *k* in *SelectKBest* class was set to a series of values that range from 5 to 150 with interval 5.

### Model training

#### Logistic regression

Logistic regression, or logit model has been widely used in bioinformatics for solving a number of classification tasks [44–46]. The goal of logistic regression is to find the best biologically reasonable model to describe the relationship between the dichotomous outcome and a set of explanatory variables. Here, the *LogisticRegressionCV* class in the Python module ‘*sklearn.linear_model*’ was used to build the logit model. When training the logit model, the fivefold cross-validator was used to automatically select the best hyperparameters, and the maximum number of iterations of the optimization algorithm was set to 5000. The “balanced” mode was used to adjust weights inversely proportional to class frequencies in the training dataset.

#### K-nearest neighbors

KNN is simple and robust algorithm, which predicts new sample by a plurality vote of its closest *k* samples from the training dataset. Here, the *KNeighborsClassifier* class in the Python module ‘*sklearn.neighbors*’ was used to build the KNN classification model. The parameter *k* is important for the performance of KNN, and it was commonly set to the square root of the number of training samples. However, our datasets are imbalanced, if *k* was chosen with this empirical rule, the neighbors would contain more negative samples, reducing the prediction possibility of true positives. Thus, *k* was set to 20 according to the number of positive samples. The *‘weights*’ was set to ‘*distance*’, and other parameters were set to default values.

#### Support vector machine

As one of the most widely used learning model, support vector machine has been applied to solve various classification problems [47–49]. It transforms the input data to higher dimension space with kernel functions, and then constructs a hyperplane to classify two types of samples. The larger the distance between the hyperplane to the nearest samples, the better the separation performance of the classifier achieves. In this paper, SVM with the radial basis function kernel was implemented using the *SVC* class in the Python module ‘*sklearn.svm*’. The kernel type of ‘*rbf*’ was used, and the probability estimates were enabled. Considering the imbalance of the training dataset, the ‘balanced’ mode was selected for improving the classification performance.

#### Random forest

Random forest is a widely used ensemble learning (ML) method that consists of a multitude of decision trees and outputs the class with the most votes from the individual trees. Here, the RF model with 100 decision trees was implemented in Python with the *RandomForestClassifier* class from the ‘*sklearn.ensemble*’ module. When training the RF model, the bootstrap samples were used to build decision trees, and the out-of-bag samples were chosen to estimate the generalization accuracy. The ‘*balanced*’ mode was used to adjust weights inversely proportional to the class frequencies in the training dataset.

#### Multi-layer perceptron

Multi-layer perceptron is a class of feedforward artificial neural network model, which consists of the input, output, and hidden layers. MLP can solve problems which are not linearly separable in the higher dimensional feature space. Here, the MLP model with two hidden layers was built using the *MLPClassifier* class in the python module ‘*sklearn.neural_network*’. The ‘*lbfgs*’ solver was used for weight optimization, and the random state for weight initialization was set to 1. Based on the number of features, the number of neurons in the 1^st^ and 2^nd^ hidden layer was set to 100 and 50, respectively. To ensure the convergence, the maximum number of iterations was set to 1000.

### Performance measures

Overall accuracy, specificity, recall, precision, F1-score, and Matthew’s correlation coefficient (MCC) were used to measure the performance for identifying pHis sites, and they are defined as follows:

4 $$Specificity = \frac{TN}{{TN + FP}}$$

5 $$Recall = \frac{TP}{{TP + FN}}$$

6 $$Precision = \frac{TP}{{TP + FP}}$$

7 $$F1 - score = \frac{2 \times Recall \times Precision}{{Recall + Precision}}$$

8 $$Accuracy = \frac{TP + TN}{{TN + FP + TP + FN}}$$

9 $$MCC = \frac{TP \times TN - FP \times FN}{{\sqrt {\left( {TP + FP} \right)\left( {TN + FN} \right)\left( {TP + FN} \right)\left( {TN + FP} \right)} }}$$

TP and TN are the number of correctly predicted pHis sites and non-pHis sites, respectively. FP and FN are the number of incorrectly predicted pHis sites and non-pHis sites, respectively.

Receiver operating characteristic (ROC curve) created by plotting the true positive rate (TPR) against the false positive rate (FPR) at various threshold values was used to visualize the predictive performance for both pHis and non-pHis sites. Precision-recall (PR) curve constructed by plotting the precision against the recall at a variety of thresholds was used to visualized the predictive performance for the pHis sites. Area under the ROC curve (AUC) is used to summarize its performance as a single number. All the performance values were calculated using the ‘metrics’ class in the Python module ‘scikit-learn’.

## Supplementary Information

**Additional file 1: Table S1.** The number of features with different window sizes. **Table S2.** The optimal feature number for different combinations of window size and model on the eukaryotic dataset. **Table S3.** The optimal feature number for different combinations of window size and model on the prokaryotic dataset. **Table S4.** The confusion matrices of PROSPECT on the eukaryotic and prokaryotic testing datasets. **Table S5.** The features used in the eukaryotic and prokaryotic model in pHisPred. **Table S6.** The confusion matrices of pHisPred on the eukaryotic and prokaryotic testing datasets.

## Data Availability

pHisPred with benchmark dataset is available at https://github.com/xiaofengsong/pHisPred, and is free for non-commercial academic use.

## Abbreviations

PTM: Post-translational modification
TCS: Two-component regulatory systems
PTS: Phosphotransferase system
Ser: Serine
Thr: Threonine
Tyr: Tyrosine
His: Histidine
pHis: Phosphohistidine
GO: Gene ontology
2D: Secondary
LR: Logistic regression
SVM: Support vector machine
RF: Random forest
MLP: Multi-layer perceptron
KNN: K-nearest neighbors
ROC: Receiver operating characteristic
AUC: Area under curve
PR: Precision-recall

## References

[1] Ardito F, Giuliani M, Perrone D, Troiano G, Muzio LL (2017). The crucial role of protein phosphorylation in cell signaling and its use as targeted therapy (review). Int J Mol Med.

[2] Fuhs SR, Hunter T (2017). pHisphorylation: the emergence of histidine phosphorylation as a reversible regulatory modification. Curr Opin Cell Biol.

[3] Potel CM, Lin M-H, Heck AJR, Lemeer S (2018). Widespread bacterial protein histidine phosphorylation revealed by mass spectrometry-based proteomics. Nat Methods.

[4] Adam K, Hunter T (2018). Histidine kinases and the missing phosphoproteome from prokaryotes to eukaryotes. Lab Invest.

[5] Kalagiri R, Stanfield RL, Meisenhelder J, Clair JJL, Fuhs SR, Wilson IA (2021). Structural basis for differential recognition of phosphohistidine-containing peptides by 1-pHis and 3-pHis monoclonal antibodies. PNAS.

[6] Attwood PV, Piggott MJ, Zu XL, Besant PG (2007). Focus on phosphohistidine. Amino Acids.

[7] Abriata LA, Albanesi D, Dal Peraro M, de Mendoza D (2017). Signal sensing and transduction by histidine kinases as unveiled through studies on a temperature sensor. Acc Chem Res.

[8] Adam K, Ning J, Reina J, Hunter T (2020). NME/NM23/NDPK and histidine phosphorylation. Int J Mol Sci.

[9] Attwood PV, Muimo R (2018). The actions of NME1/NDPK-A and NME2/NDPK-B as protein kinases. Lab Invest.

[10] Fuhs SR, Meisenhelder J, Aslanian A, Ma L, Zagorska A, Stankova M (2015). Monoclonal 1- and 3-phosphohistidine antibodies: new tools to study histidine phosphorylation. Cell.

[11] Boissan M, Montagnac G, Shen Q, Griparic L, Guitton J, Romao M (2014). Membrane trafficking. Nucleoside diphosphate kinases fuel dynamin superfamily proteins with GTP for membrane remodeling. Science.

[12] Conery AR, Sever S, Harlow E (2010). Nucleoside diphosphate kinase Nm23-H1 regulates chromosomal stability by activating the GTPase dynamin during cytokinesis. Proc Natl Acad Sci U S A.

[13] Besant PG, Attwood PV (2005). Mammalian histidine kinases. Biochim Biophys Acta.

[14] Hindupur SK, Colombi M, Fuhs SR, Matter MS, Guri Y, Adam K (2018). The protein histidine phosphatase LHPP is a tumour suppressor. Nature.

[15] Liu Y, Xia C, Fan Z, Jiao F, Gao F, Xie Y (2020). Novel two-dimensional MoS_2_–Ti^4+^ nanomaterial for efficient enrichment of phosphopeptides and large-scale identification of histidine phosphorylation by mass spectrometry. Anal Chem.

[16] Vander Heiden MG, Locasale JW, Swanson KD, Sharfi H, Heffron GJ, Amador-Noguez D (2010). Evidence for an alternative glycolytic pathway in rapidly proliferating cells. Science.

[17] Boyer PD, Deluca M, Ebner KE, Hultquist DE, Peter JB (1962). Identification of phosphohistidine in digests from a probable intermediate of oxidative phosphorylation. J Biol Chem.

[18] Marmelstein AM, Moreno J, Fiedler D (2017). Chemical approaches to studying labile amino acid phosphorylation. Top Curr Chem (Z).

[19] Gonzalez-Sanchez M-B, Lanucara F, Helm M, Eyers CE (2013). Attempting to rewrite History: challenges with the analysis of histidine-phosphorylated peptides. Biochem Soc Trans.

[20] Yagüe P, Gonzalez-Quiñonez N, Fernánez-García G, Alonso-Fernández S, Manteca A (2019). Goals and challenges in bacterial phosphoproteomics. Int J Mol Sci.

[21] Makwana MV, Muimo R, Jackson RF (2018). Advances in development of new tools for the study of phosphohistidine. Lab Invest.

[22] Gao Y, Lee H, Kwon OK, Cheng Z, Tan M, Kim K-T (2019). Profiling of histidine phosphoproteome in *Danio rerio* by TiO_2_ enrichment. Proteomics.

[23] Hardman G, Perkins S, Brownridge PJ, Clarke CJ, Byrne DP, Campbell AE (2019). Strong anion exchange-mediated phosphoproteomics reveals extensive human non-canonical phosphorylation. EMBO J.

[24] Potel CM, Lin M-H, Prust N, van den Toorn HWP, Heck AJR, Lemeer S (2019). Gaining confidence in the elusive histidine phosphoproteome. Anal Chem.

[25] Lapek JDJ, Tombline G, Kellersberger KA, Friedman MR, Friedman AE (2015). Evidence of histidine and aspartic acid phosphorylation in human prostate cancer cells. Naunyn Schmiedebergs Arch Pharmacol.

[26] Trost B, Kusalik A (2011). Computational prediction of eukaryotic phosphorylation sites. Bioinformatics.

[27] Xue Y, Li A, Wang L, Feng H, Yao X (2006). PPSP: prediction of PK-specific phosphorylation site with Bayesian decision theory. BMC Bioinform.

[28] Wang D, Zeng S, Xu C, Qiu W, Liang Y, Joshi T (2017). MusiteDeep: a deep-learning framework for general and kinase-specific phosphorylation site prediction. Bioinformatics.

[29] Luo F, Wang M, Liu Y, Zhao X-M, Li A (2019). DeepPhos: prediction of protein phosphorylation sites with deep learning. Bioinformatics.

[30] Wang C, Xu H, Lin S, Deng W, Zhou J, Zhang Y (2020). GPS 5.0: an update on the prediction of kinase-specific phosphorylation sites in proteins. Genom Proteom Bioinform.

[31] Awais M, Hussain W, Khan YD, Rasool N, Khan SA, Chou K-C (2019). iPhosH-PseAAC: identify phosphohistidine sites in proteins by blending statistical moments and position relative features according to the Chou’s 5-step rule and general pseudo amino acid composition. IEEE/ACM Trans Comput Biol Bioinform.

[32] UniProt Consortium (2021). UniProt: the universal protein knowledgebase in 2021. Nucleic Acids Res.

[33] Huang K-Y, Lee T-Y, Kao H-J, Ma C-T, Lee C-C, Lin T-H (2019). dbPTM in 2019: exploring disease association and cross-talk of post-translational modifications. Nucleic Acids Res.

[34] Chen Z, Zhao P, Li F, Leier A, Marquez-Lago TT, Webb GI (2020). PROSPECT: a web server for predicting protein histidine phosphorylation sites. J Bioinform Comput Biol.

[35] Vacic V, Iakoucheva LM, Radivojac P (2006). Two sample logo: a graphical representation of the differences between two sets of sequence alignments. Bioinformatics.

[36] Fiorini N, Lipman DJ, Lu Z (2017). Towards PubMed 2.0. Elife.

[37] Hornbeck PV, Zhang B, Murray B, Kornhauser JM, Latham V, Skrzypek E (2015). PhosphoSitePlus, 2014: mutations, PTMs and recalibrations. Nucleic Acids Res.

[38] Ullah S, Lin S, Xu Y, Deng W, Ma L, Zhang Y (2016). dbPAF: an integrative database of protein phosphorylation in animals and fungi. Sci Rep.

[39] Yu K, Zhang Q, Liu Z, Zhao Q, Zhang X, Wang Y (2019). qPhos: a database of protein phosphorylation dynamics in humans. Nucleic Acids Res.

[40] Blom N, Kreegipuu A, Brunak S (1998). PhosphoBase: a database of phosphorylation sites. Nucleic Acids Res.

[41] National Center for Biotechnology Information (NCBI). Documentation of the BLASTCLUST-algorithm.

[42] Li W, Godzik A (2006). Cd-hit: a fast program for clustering and comparing large sets of protein or nucleotide sequences. Bioinformatics.

[43] Chen Z, Zhao P, Li F, Leier A, Marquez-Lago TT, Wang Y (2018). iFeature: a Python package and web server for features extraction and selection from protein and peptide sequences. Bioinformatics.

[44] Zhao J, Song X, Wang K (2016). lncScore: alignment-free identification of long noncoding RNA from assembled novel transcripts. Sci Rep.

[45] Zhao J, Wu J, Xu T, Yang Q, He J, Song X (2018). IRESfinder: Identifying RNA internal ribosome entry site in eukaryotic cell using framed k-mer features. J Genet Genom.

[46] Wang L, Park HJ, Dasari S, Wang S, Kocher J-P, Li W (2013). CPAT: coding-potential assessment tool using an alignment-free logistic regression model. Nucleic Acids Res.

[47] Kong L, Zhang Y, Ye Z-Q, Liu X-Q, Zhao S-Q, Wei L (2007). CPC: assess the protein-coding potential of transcripts using sequence features and support vector machine. Nucleic Acids Res.

[48] Meng C, Jin S, Wang L, Guo F, Zou Q (2019). AOPs-SVM: a sequence-based classifier of antioxidant proteins using a support vector machine. Front Bioeng Biotechnol.

[49] Huang S, Cai N, Pacheco PP, Narandes S, Wang Y, Xu W (2017). Applications of support vector machine (SVM) learning in cancer genomics. Cancer Genom Proteom.

