# Supplementary material for: pHisPred: a tool for the identification of histidine phosphorylation sites by integrating amino acid patterns and properties
Source: BMC Bioinformatics. 2022 Sep 28;23(Suppl 3):399. doi: 10.1186/s12859-022-04938-x (PMC9520798; doi:10.1186/s12859-022-04938-x)
Supplement: Supplementary file 1 — Additional file 1: Table S1. The number of features with different window sizes. Table S2. The optimal feature number for different combinations of window size and model on the eukaryotic dataset. Table S3. The optimal feature number for different combinations of window size and model on the prokaryotic dataset. Table S4. The confusion matrices of PROSPECT on the eukaryotic and prokaryotic testing datasets. Table S5. The features used in the eukaryotic and prokaryotic model in pHisPred. Table S6. The confusion matrices of pHisPred on the eukaryotic and prokaryotic testing datasets. [file 12859_2022_4938_MOESM1_ESM.docx]

Table S1. The number of features with different window sizes

| **Feature type** | **Window size** | | | | |
| --- | --- | --- | --- | --- | --- |
|  | **21** | **25** | **31** | **35** | **41** |
| AAC | 20 | 20 | 20 | 20 | 20 |
| EAAC | 340 | 420 | 540 | 620 | 740 |
| CKSAAP | 2400 | 2400 | 2400 | 2400 | 2400 |
| DPC | 400 | 400 | 400 | 400 | 400 |
| TPC | 8000 | 8000 | 8000 | 8000 | 8000 |
| GAAC | 5 | 5 | 5 | 5 | 5 |
| EGAAC | 85 | 105 | 135 | 155 | 185 |
| CKSAAGP | 150 | 150 | 150 | 150 | 150 |
| GDPC | 25 | 25 | 25 | 25 | 25 |
| GTPC | 125 | 125 | 125 | 125 | 125 |
| CTD-C | 39 | 39 | 39 | 39 | 39 |
| CTD-D | 195 | 195 | 195 | 195 | 195 |
| CTD-T | 39 | 39 | 39 | 39 | 39 |
| C-Triad | 343 | 343 | 343 | 343 | 343 |
| CKS-Triad | 1715 | 343 | 343 | 343 | 343 |
| SOC-Number | 20 | 20 | 30 | 30 | 40 |
| QS-Order | 60 | 60 | 70 | 70 | 80 |
| PAAC | 30 | 30 | 35 | 35 | 40 |
| APAAC | 40 | 40 | 50 | 50 | 60 |
| NM-Broto | 80 | 80 | 120 | 120 | 160 |
| Moran | 80 | 80 | 120 | 120 | 160 |
| Geary | 80 | 80 | 120 | 120 | 160 |
| Binary | 420 | 500 | 620 | 700 | 820 |
| AA-index | 11151 | 13275 | 16461 | 18585 | 21771 |
| BLOSUM62 | 420 | 500 | 620 | 700 | 820 |
| Z-scale | 105 | 125 | 155 | 175 | 205 |
| Total | 26367 | 27399 | 31160 | 33564 | 37325 |

Table S2. The optimal feature number for different combinations of window size and model on the eukaryotic dataset.

| **Model** | **Window size** | **Optimal feature number** | ***AUC*** | ***F1-score*** | **Mean** |
| --- | --- | --- | --- | --- | --- |
| LR | 21 | 70 | 0.7491 | 0.3386 | 0.54385 |
|  | 25 | 150 | 0.7827 | 0.3231 | 0.5529 |
|  | 31 | 150 | 0.7868 | 0.3434 | 0.5651 |
|  | 35 | 120 | 0.8216 | 0.3683 | 0.59495 |
|  | 41 | 110 | 0.7815 | 0.3218 | 0.55165 |
| KNN | 21 | 70 | 0.6511 | 0.2207 | 0.4359 |
|  | 25 | 50 | 0.6749 | 0.2632 | 0.46905 |
|  | 31 | 50 | 0.6895 | 0.0994 | 0.39445 |
|  | 35 | 70 | 0.6794 | 0.2037 | 0.44155 |
|  | 41 | 30 | 0.6307 | 0.2815 | 0.4561 |
| SVM | 21 | 100 | 0.7946 | 0.3739 | 0.58425 |
|  | 25 | 150 | 0.8209 | 0.3966 | 0.60875 |
|  | 31 | 140 | 0.8139 | 0.3958 | 0.60485 |
|  | 35 | 140 | 0.814 | 0.3486 | 0.5813 |
|  | 41 | 130 | 0.7846 | 0.3214 | 0.553 |
| RF | 21 | 20 | 0.6211 | 0.3828 | 0.50195 |
|  | 25 | 70 | 0.73 | 0.1594 | 0.4447 |
|  | 31 | 30 | 0.5709 | 0.2378 | 0.40435 |
|  | 35 | 20 | 0.5913 | 0.2816 | 0.43645 |
|  | 41 | 50 | 0.7354 | 0.1193 | 0.42735 |
| MLP | 21 | 110 | 0.6468 | 0.2842 | 0.4655 |
|  | 25 | 120 | 0.6903 | 0.3398 | 0.51505 |
|  | 31 | 150 | 0.6438 | 0.3262 | 0.485 |
|  | 35 | 120 | 0.6824 | 0.3022 | 0.4923 |
|  | 41 | 90 | 0.6356 | 0.3317 | 0.48365 |

The ‘Mean’ denotes the mean value of *AUC* and *F1-score*.

Table S3. The optimal feature number for different combinations of window size and model on the prokaryotic dataset.

| **Model** | **Window size** | **Optimal feature number** | ***AUC*** | ***F1-score*** | **Mean** |
| --- | --- | --- | --- | --- | --- |
| LR | 21 | 150 | 0.7902 | 0.4196 | 0.6049 |
|  | 25 | 150 | 0.7804 | 0.4481 | 0.61425 |
|  | 31 | 150 | 0.7785 | 0.4522 | 0.61535 |
|  | 35 | 90 | 0.7631 | 0.4133 | 0.5882 |
|  | 41 | 80 | 0.764 | 0.4193 | 0.59165 |
| KNN | 21 | 80 | 0.6936 | 0.31 | 0.5018 |
|  | 25 | 80 | 0.7206 | 0.2988 | 0.5097 |
|  | 31 | 80 | 0.6768 | 0.334 | 0.5054 |
|  | 35 | 70 | 0.7021 | 0.2819 | 0.492 |
|  | 41 | 110 | 0.6983 | 0.2583 | 0.4783 |
| SVM | 21 | 140 | 0.7852 | 0.4262 | 0.6057 |
|  | 25 | 100 | 0.7957 | 0.4583 | 0.627 |
|  | 31 | 150 | 0.7799 | 0.4609 | 0.6204 |
|  | 35 | 150 | 0.7707 | 0.4267 | 0.5987 |
|  | 41 | 140 | 0.764 | 0.4201 | 0.59205 |
| RF | 21 | 10 | 0.6749 | 0.2968 | 0.48585 |
|  | 25 | 10 | 0.6925 | 0.303 | 0.49775 |
|  | 31 | 130 | 0.7782 | 0.2422 | 0.5102 |
|  | 35 | 40 | 0.6964 | 0.1898 | 0.4431 |
|  | 41 | 50 | 0.7109 | 0.1586 | 0.43475 |
| MLP | 21 | 150 | 0.7292 | 0.4 | 0.5646 |
|  | 25 | 100 | 0.7373 | 0.397 | 0.56715 |
|  | 31 | 120 | 0.7018 | 0.4036 | 0.5527 |
|  | 35 | 120 | 0.6979 | 0.4105 | 0.5542 |
|  | 41 | 150 | 0.7162 | 0.3634 | 0.5398 |

The ‘Mean’ denotes the mean value of *AUC* and *F1-score*.

Table S4. The features used in the eukaryotic and prokaryotic model in pHisPred.

| Group | Eukaryotic features | Prokaryotic features |
| --- | --- | --- |
| AAC | -- | K |
| AAINDEX | SeqPos.1.ANDN920101  SeqPos.1.BUNA790102  SeqPos.1.PALJ810107  SeqPos.1.VINM940104  SeqPos.2.ANDN920101  SeqPos.2.BUNA790102  SeqPos.2.FASG760101  SeqPos.2.KARP850102  SeqPos.2.LEVM760103  SeqPos.2.LEVM780101  SeqPos.2.OOBM770105  SeqPos.2.PALJ810109  SeqPos.2.PRAM900102  SeqPos.2.RACS820108  SeqPos.2.ONEK900101  SeqPos.2.VINM940101  SeqPos.2.VINM940102  SeqPos.2.VINM940103  SeqPos.2.VINM940104  SeqPos.2.GEOR030101  SeqPos.2.GEOR030102  SeqPos.2.GEOR030104  SeqPos.2.GEOR030106  SeqPos.2.GEOR030107  SeqPos.2.GEOR030108  SeqPos.4.FAUJ880105  SeqPos.4.LEVM760103  SeqPos.4.PONP800106  SeqPos.4.SNEP660101  SeqPos.4.TANS770103  SeqPos.4.KIMC930101  SeqPos.14.GEOR030107  SeqPos.16.CHOC760103  SeqPos.16.CHOP780204  SeqPos.16.CHOP780207  SeqPos.16.GEIM800104  SeqPos.16.GEIM800107  SeqPos.16.GRAR740102  SeqPos.16.ISOY800101  SeqPos.16.LEVM780103  SeqPos.16.LIFS790101  SeqPos.16.OOBM770104  SeqPos.16.PONP800101  SeqPos.16.QIAN880111  SeqPos.16.QIAN880119  SeqPos.16.QIAN880130  SeqPos.16.RACS820101  SeqPos.16.RACS820104  SeqPos.16.RACS820107  SeqPos.16.RICJ880115  SeqPos.16.ROBB760110  SeqPos.16.TANS770102  SeqPos.16.AURR980102  SeqPos.16.AURR980107  SeqPos.16.MUNV940104  SeqPos.16.FUKS010110  SeqPos.16.COSI940101  SeqPos.16.WILM950102  SeqPos.16.WOLR790101  SeqPos.16.GUYH850105  SeqPos.16.JACR890101  SeqPos.24.FASG760101  SeqPos.24.OOBM770105  SeqPos.28.GEOR030107 | SeqPos.1.MAXF760102  SeqPos.1.WIMW960101  SeqPos.8.EISD860102  SeqPos.8.YUTK870103  SeqPos.8.YUTK870104  SeqPos.13.FAUJ880111  SeqPos.13.RICJ880114  SeqPos.13.COWR900101  SeqPos.14.PALJ810116  SeqPos.15.QIAN880139  SeqPos.15.RICJ880114  SeqPos.16.CHOC760104  SeqPos.16.CHOP780204  SeqPos.16.CHOP780216  SeqPos.16.CIDH920103  SeqPos.16.EISD860101  SeqPos.16.FAUJ880102  SeqPos.16.FAUJ880108  SeqPos.16.GEIM800107  SeqPos.16.KARP850103  SeqPos.16.NAKH900102  SeqPos.16.NAKH900104  SeqPos.16.NAKH900108  SeqPos.16.NISK860101  SeqPos.16.QIAN880109  SeqPos.16.QIAN880116  SeqPos.16.QIAN880117  SeqPos.16.QIAN880118  SeqPos.16.QIAN880120  SeqPos.16.QIAN880121  SeqPos.16.QIAN880122  SeqPos.16.RACS820101  SeqPos.16.RACS820107  SeqPos.16.SNEP660103  SeqPos.16.SWER830101  SeqPos.16.TANS770105  SeqPos.16.AURR980102  SeqPos.16.AURR980116  SeqPos.16.COSI940101  SeqPos.16.PUNT030101  SeqPos.16.ZHOH040102  SeqPos.16.MIYS990103  SeqPos.16.MIYS990104  SeqPos.16.MIYS990105  SeqPos.17.CHAM820101  SeqPos.17.CHAM830108  SeqPos.17.FAUJ880106  SeqPos.17.LEVM760107  SeqPos.17.PRAM820102  SeqPos.17.QIAN880129  SeqPos.20.ROSG850101  SeqPos.22.FAUJ880111 |
| APAAC | Pc1.Q | -- |
| BINARY | BINARY.F180  BINARY.F249  BINARY.F269 | BINARY.F229  BINARY.F249  BINARY.F269  BINARY.F289  BINARY.F492 |
| BLOSUM62 | -- | blosum62.F142  blosum62.F229  blosum62.F249  blosum62.F289 |
| CKSAAGP | alphaticr.postivecharger.gap0  uncharger.uncharger.gap0  alphaticr.postivecharger.gap1  uncharger.uncharger.gap2  uncharger.uncharger.gap3  postivecharger.alphaticr.gap4 | alphaticr.aromatic.gap0  alphaticr.postivecharger.gap0  alphaticr.aromatic.gap1  alphaticr.postivecharger.gap1  postivecharger.postivecharger.gap1  alphaticr.aromatic.gap2  aromatic.alphaticr.gap2  postivecharger.alphaticr.gap2  postivecharger.postivecharger.gap2  uncharger.postivecharger.gap2  postivecharger.negativecharger.gap3  uncharger.postivecharger.gap3  alphaticr.aromatic.gap4  alphaticr.postivecharger.gap4  aromatic.alphaticr.gap4  postivecharger.alphaticr.gap4  postivecharger.alphaticr.gap5  postivecharger.postivecharger.gap5 |
| CKSAAP | GK.gap0  RF.gap0  WP.gap0  FF.gap1  WK.gap1  IS.gap3  FR.gap5  GA.gap5 | HH.gap0  HH.gap1  HH.gap2  HH.gap3 |
| CTDC | hydrophobicity_ARGP820101.G2 | hydrophobicity_PRAM900101.G3  hydrophobicity_ARGP820101.G2  hydrophobicity_ARGP820101.G3  hydrophobicity_ZIMJ680101.G2  hydrophobicity_ZIMJ680101.G3  hydrophobicity_PONP930101.G3  hydrophobicity_CASG920101.G3  polarity.G1  polarity.G3  charge.G1  solventaccess.G1 |
| CTDD | -- | charge.1.residue100 |
| CTDT | -- | hydrophobicity_ARGP820101.Tr1221  hydrophobicity_ARGP820101.Tr1331  hydrophobicity_ZIMJ680101.Tr1221  hydrophobicity_ZIMJ680101.Tr1331  hydrophobicity_PONP930101.Tr1221  hydrophobicity_CASG920101.Tr1331  hydrophobicity_CASG920101.Tr2332  normwaalsvolume.Tr1331  polarity.Tr2332  polarizability.Tr1331  charge.Tr1221 |
| CTriad | g5.g2.g7  g7.g2.g7 | g5.g3.g4  g7.g5.g4 |
| DPC | GK  RF  WP | HH |
| EAAC | SW.12.H | SW.9.H  SW.10.H  SW.11.H  SW.12.H  SW.13.H  SW.14.H  SW.21.K  SW.22.K  SW.23.K  SW.24.K |
| EGAAC | -- | SW.8.postivecharger  SW.9.postivecharger  SW.10.postivecharger  SW.11.aromatic  SW.11.postivecharger  SW.12.postivecharger  SW.13.aromatic  SW.13.postivecharger  SW.21.postivecharger  SW.22.postivecharger |
| GAAC | uncharge | aromatic  postivecharge |
| GDPC | alphaticr.postivecharger  uncharger.uncharger | alphaticr.aromatic  alphaticr.postivecharger |
| GTPC | -- | alphaticr.alphaticr.aromatic |
| Geary | GCHAM820102.lag1 | -- |
| KSCTriad | g5.g2.g7.gap0  g7.g2.g7.gap0 | g5.g3.g4.gap0  g7.g5.g4.gap0 |
| Moran | MCHAM820102.lag1 | -- |
| PAAC | Xc1.Q | Xc1.R |
| QSOrder | Schneider.Xr.K | Schneider.Xr.R  Grantham.Xr.R  Grantham.Xr.H  Grantham.Xr.K |
| SOCNumber | -- | Schneider.lag5  gGrantham.lag6 |
| TPC | AER  AGA  AHF  ANG  CAV  CFC  DAI  DLH  DPV  DWP  EGA  FDR  FIS  FPR  GDW  GGA  GVT  HAW  HGE  HMN  IFD  KAT  KDI  KLP  LFI  MIK  MPN  NAL  PTK  PYL  QAN  QGI  RFA  RFS  RHG  SGK  SMF  VYC  WGK  WPL  YGS | FRR  HDH  RHT  RTP  TKF  TKP |
| ZSCALE | Pos16.ZSCALE1 | -- |

Description of each feature group can be seen in Table 1, and feature names were derived from iFeature (<https://github.com/Superzchen/iFeature>).

Table S5. The confusion matrices of pHisPred on the eukaryotic and prokaryotic testing datasets.

| Eukaryotic  testing dataset | | Predicted result | | prokaryotic  testing dataset | | Predicted result | |
| --- | --- | --- | --- | --- | --- | --- | --- |
|  |  | pHis | non-pHis |  |  | pHis | non-pHis |
| Actual  condition | pHis | 19 | 12 | Actual  condition | pHis | 51 | 21 |
|  | non-pHis | 37 | 372 |  | non-pHis | 79 | 263 |

Table S6. The confusion matrices of PROSPECT on the eukaryotic and prokaryotic testing datasets.

| Eukaryotic  testing dataset | | Predicted result | | prokaryotic  testing dataset | | Predicted result | |
| --- | --- | --- | --- | --- | --- | --- | --- |
|  |  | pHis | non-pHis |  |  | pHis | non-pHis |
| Actual  condition | pHis | 31 | 0 | Actual  condition | pHis | 21 | 0 |
|  | non-pHis | 409 | 0 |  | non-pHis | 110 | 0 |
